# 0601. An inhibitor of pro-inflammatory enzyme soluble epoxide hydrolase improves hippocampus dependent memory function after cardiac arrest and cardio-pulmonary resuscitation in mice

**DOI:** 10.1186/2197-425X-2-S1-P42

**Published:** 2014-09-26

**Authors:** T Fujiyoshi, S Hoka

**Affiliations:** Kyushu University, Department of Anesthesiology, Fukuoka, Japan

## Introduction

Promotion and improvement of cardiopulmonary resuscitation (CPR) technique contributes to increase of the survivors from unexpected cardiac arrest (CA). However, many patients have some neurological deficits, because of few effective treatment to improve the outcome. Hippocampus damage causes a memory dysfunction which suffers the survivors. Microglia activation caused neuron damage following brain stroke and inhibition of pro-inflammatory soluble epoxide hydrolase (sEH) reduced the infarction size in partial ischemia mice model.

## Objectives

We investigated whether therapeutic inhibition of sEH suppresses neuronal damage and improve memory dysfunction.

## Methods

Under isoflurane anesthesia, male adult C57Bl/6 mice were orally intubated and were connected to the ventilator. A jugular vein catheter, thermometer and electrocardiography (EKG) was placed. CA was induced by potassium chloride injection. When cardiac arrest was confirmed by EKG, isoflurane and mechanical ventilation were disconnected. Brain temperature was maintained at 37.5 ± 0.5 °C and rectal temperature is decreased to 28 ± 0.5 °C during CA. Mechanical ventilation was restarted 30 seconds before CPR and CPR, chest compression and epinephrine administration, was initiated at 8 minutes after CA onset. The sEH inhibitor 4-phenylchalcone oxide (4-PCO, 5 mg/kg ip) or vehicle was injected at 5 minutes after CACPR and repeated daily. Neuro-scoring examination was performed 3 days after CAPR. Surviving CA1 hippocampal neurons were counted 1, 3 or 10 days and microglia activation was assessed by immunostaining for the activation marker Mac-2 at 1, 3, and 10 days after CACPR Delay fear conditioning was tested 10 days after CA/CPR.RESULTS; Succeeded CPR rate was 86.4 % and 65.9 % of CACPR mice survived for 10 days after CACPR. CACPR mice showed some neurological deficits on day 3, whereas sham-operated mice showed no deficits. Delayed neuron death appeared on day 3 and persisted 10 days after CACPR in hippocampus CA1 area of CACPR mice. Microglia activated on day 1, migrated to the ischemia-induced damaged hippocampus area on day 3 and persisted on day 10 in CACPR mice. In CACPR mice, 4-PCO activated microglia on day 3, but surprisingly, 4-PCO reduced damaged neurons on day 3, not on day 10. CACPR mice showed reduced freezing to context, not to cued and CACPR mice treated by 4-PCO showed more freezing to context, significantly. This result of fear conditioning test indicated that CACPR caused hippocampus dependent memory deficit, which was improved by 4-PCO treatment.

## Conclusions

Experimental CACPR caused hippocampal neuronal damage, microglial activation, and memory deficit in mice. Inhibition of sEH activated microglia but protected ischemia-induced neurons and improved memory function in hippocampus in mice. The inhibition of sEH will be a key of new therapeutic approach to global ischemia-induced damaged brain.

